# Adductor Muscles Strength and Strength Asymmetry as Risk Factors for Groin Injuries among Professional Soccer Players: A Prospective Study

**DOI:** 10.3390/ijerph17144946

**Published:** 2020-07-09

**Authors:** Goran Markovic, Nejc Šarabon, Jelena Pausic, Vedran Hadžić

**Affiliations:** 1 Faculty of Kinesiology, University of Zagreb, 10110 Zagreb, Croatia; 2 Motus Melior Ltd., Hektorovićeva 2, 10000 Zagreb, Croatia; 3 Faculty of Health Sciences, University of Primorska, Polje 42, 6310 Izola, Slovenia; nejc.sarabon@fvz.upr.si; 4 S2P, Science to Practice, Ltd., Laboratory for Motor Control and Motor Behavior, Tehnološki Park 19, 1000 Ljubljana, Slovenia; 5 Faculty of Kinesiology, University of Split, Ul. Nikole Tesle 6, 21000 Split, Croatia; jelenap@kifst.hr; 6 Faculty of Sport, University of Ljubljana, Gortanova ul. 22, 1000 Ljubljana, Slovenia; vedran.hadzic@fsp.uni-lj.si

**Keywords:** injury prevention, dynamometry, hip and groin

## Abstract

The aim of this study was to prospectively examine the association between isometric hip adductor strength and between-limb strength asymmetry to groin injuries in male professional soccer players. Isometric hip adductor strength and between-limb strength asymmetry of 45 professional outfield soccer players from three First Division teams were tested during the 2017/2018 preseason. Players were then monitored throughout the 2017/2018 season for groin injuries. Ten groin injuries were recorded. When compared with uninjured players, players who sustained groin injury had significantly lower strength of respective muscle groups and significantly higher between-limb strength asymmetries (all *p* < 0.05; ES = 1.16 and 0.88; mean % difference = 26% and 51%). Isometric hip adductor strength had a significant inverse relationship with the incidence of occurring groin injuries (*p* = 0.016). No significant relationship between hip adductor strength asymmetry and the incidence of future groin injury was observed (*p* = 0.09). Finally, players’ age and previous groin injury were not significantly associated with the incidence of future groin injuries (all *p* > 0.05). These results generally suggest that isometric adductor strength is a significant predictor of future groin injuries in men’s professional football; however, due to the relatively low sample size, further studies are required.

## 1. Introduction

Groin injuries are among the most common injuries in men’s soccer, accounting for 14% to 19% of all injuries [1,2]. Recent epidemiological data suggest that 20–25% of all players sustain a time-loss groin injury in a season [3,4], with recurrence injury rate ranging from 14% [3] to 30% [5]. Note, however, that the traditional time-loss measure captures only up to one third of all groin problems in male soccer players [6,7]. Hence, groin problems represent a significant health and performance burden in men’s soccer. In order to successfully implement preventive measures to reduce the risk of injury incidence, the identification of the risk factors associated with the occurrence of groin injury and their monitoring is essential [8,9].

Among numerous non-modifiable and modifiable risk factors associated with groin injuries in sports [8,10,11], two modifiable factors, i.e., hip adductor strength and hip range of motion, have received particular scientific interest in men’s soccer [12,13,14,15,16,17]. Regarding hip range of motion, previous prospective studies have generally shown that hip range of motion tests are not associated with an increased risk of groin injury in men’s soccer [14,15,17]. For isometric hip adductor strength, the results are conflicting; while some authors have reported significant associations between isometric hip adductor strength and groin injury risk in men’s soccer [14,16], others failed to establish such associations [12,17].

Notably, there is a rather large variability in testing procedure used for adductor strength measurement among these studies. Bakken et al. [12], Moreno-Pérez et al. [16], and Mosler et al. [17] used a bilateral adductor squeeze test with the hand-held dynamometer being placed between the bent knees and only Moreno-Pérez et al. [16] reported significant association between isometric adductor strength and groin injury risk. Furthermore, Engebretsen et al. [14] and Mosler et al. [17] measured isometric adductor strength with the hand-held dynamometer placed above the medial malleoli of the extended leg and reported conflicting findings. Finally, Bourne et al. [13] recently combined the two testing approaches by using a novel externally fixed dynamometer device and prospectively showed that the principal component that represents strength of both hip abductor and adductor muscles measured in two positions is a significant risk factor for groin injury in male soccer players.

When considering the common injury mechanism(s) for acute groin injuries in soccer (i.e., kicking and changing direction [18]), it seems logical that the measurement of players’ hip adductor strength as the potential risk factor for groin injuries should include both uni- and bi-articular hip adductor muscles. Indeed, it has recently been shown that the gracilis (bi-articular hip adductor) experiences considerable forces and stress during soccer inside passing [19]. Furthermore, there is increasing evidence of structural in-series continuity between skeletal muscles in the lower extremity [20]. All this suggests that the hip adductor strength of soccer players should be tested with long lever arm, i.e., by placing the dynamometer at the ankle level. Notably, Bourne et al. [13] recently reported that only about 50% of common variance is shared between hip adduction strength tests measured at knee and ankle levels, respectively.

Another potential problem is related to the limitations of frequently used hand-held dynamometry for measurement of hip adductor strength. Thorborg et al. [21] showed that this testing method is susceptible to error without external fixation and may be confounded by inter-tester bias. The authors suggested external fixation of the dynamometer as a solution for significant inter-observed systematic bias and confirmed the validity of this approach experimentally [22]. Of all cited prospective studies, only Bourne et al. [13] used an externally fixed dynamometer for the measurement of hip adductor strength at the ankle level; however, by using principal component analysis, the authors reduced hip adductor and abductor strength measured at both the ankle and knee levels (i.e., four strength variables) to a single variable (principal component), and associated this variable with groin injuries in soccer. Hence, our knowledge about hip adductor strength as a risk factor for groin injuries in men’s soccer is still limited.

The purpose of this study was to prospectively examine the association between isometric hip adductor strength (measured with an externally fixed dynamometer at the ankle level) and groin injuries in male professional soccer players. Since only one previous prospective study considered between-limb strength asymmetry of hip adductors as a possible risk factor for groin injury in soccer (Bourne et al. [13]), we also included this variable in the study.

## 2. Materials and Methods

### 2.1. Participants and Study Design

This prospective cohort study was completed during the preseason (June–July 2017) and in-season period (September 2017–May 2018) of the 2017/2018 Croatian Major League competition. The sample consisted of 45 healthy, male, professional outfield soccer players (mean ± SD; age: 22.8 ± 3.5 years; height: 1.82 ± 0.06 m; mass: 78.0 ± 7.0 kg), recruited from 3 Croatian Major League teams. Only players (i.e., 45 out of 68 players) who were injury-free 6 weeks before the start of preseason, and who played at least 45 min in more than 20 professional matches in the respective season, were included to decrease the influence of training and match exposures [23,24]. Thirteen out of 45 players were members of their respective national teams or U21 national teams. During the whole season, all three soccer teams performed resistance and injury-prevention training focused on strengthening the most injured muscle groups in soccer (hamstrings, quadriceps, hip adductors, and calves) and balance-proprioception work for the hip, knee, and ankle joints. All players gave their informed consent for inclusion before they participated in the study. The study was conducted in accordance with the Declaration of Helsinki, and the protocol was approved by the Ethics Committee of the Faculty of Kinesiology, University of Zagreb (2017-ZUID-15).

### 2.2. Injury Registration

Club medical staff completed a retrospective injury questionnaire that detailed each athlete’s history of groin injuries/pain in the past 12 months. Furthermore, club medical staff recorded all injuries during the competitive season, including groin injuries. Specifically, they completed a standardized injury report form that included: the date of injury; training or match injury occurrence; date of return to training and competition; side of injury (preferred—kicking, or non-preferred—non-kicking); injury location (adductor-related; iliopsoas-related; inguinal-related; pubic-related; hip-related; other); injury severity (grade 1–3); and whether it was a first-time or recurrent injury. All groin injuries were diagnosed clinically by the experienced club doctors, and subsequently confirmed by MRI scan or diagnostic ultrasound.

As suggested by Engebretsen et al. [14], “an injury was defined as any physical complaint sustained by a player that made him seek medical assistance and that resulted from a soccer match or soccer training, forcing him to miss or being unable to take full part in future soccer training or match play (“time-loss” injury).” For the purpose of the present article, a groin injury was defined as an “injury located to the hip joint or surrounding soft tissues or at the junction between the anteromedial part of the thigh, including the proximal part of the adductor muscle bellies, pubic symphysis, and the lower abdomen” [17]. Injuries were classified into 3 severity categories: minor (1–7 days), moderate (8–28 days), and major (>28 days) [25].

### 2.3. Strength Testing

Strength testing of adductor muscles was performed at the beginning of the 2017/2018 pre-season. The body mass and body height of each player were measured to the nearest 0.1 kg and 0.5 cm using a calibrated medical scale and stadiometer (SECA 284; Seca, Hamburg, Germany). Leg length, determined using previously described procedures [26], was used to convert the force measurements to torque.

**Figure 1:**
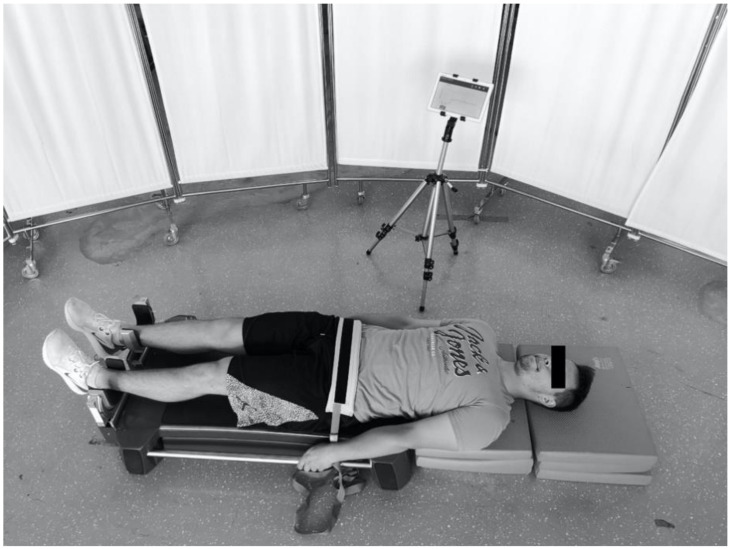
Position of the participant during isometric adductor strength testing.

After a standardized warm-up (running 5 min at a comfortable pace, 2 min of dynamic stretching, 2 min of skipping and footwork drills, 10 body-weight squats, 10 sit-ups, 10 forward lunges, 5 short sprint accelerations, and 1 set of three 5-second submaximal ball squeezes with the feet), players performed bilateral isometric hip adductor strength testing using externally fixed dynamometry (Figure 1), similar to the one recently described by Bourne et al. [13] and O’Brien et al. [27]. In brief, players were required to lie in a supine position with their knees and hips fully extended. They placed their shanks (lower legs) onto “U” shaped padded aluminium braces, which were attached to uniaxial load cells (FL34-100 kg; Forsentek Co., Shenzhen, China). Load cells were positioned perpendicular to the medial part of the tibia. The distance between the players’ feet was adjusted to their hip width, while the distance between the mid-part of the aluminium brace and their medial malleoli was set to 5 cm. Players were asked to complete a maximum repetition, pushing their distal shanks against the pads for 5 s, providing a measure of force (N) for left and right limbs. During testing, players were allowed to hold the side of the plinths. The hip adductor strength test was performed three times, with a 45-second break between trials. Force signals, sampled at a sampling rate of 1000 Hz, were preprocessed with a moving average filter using a 10-ms window. The peak force value during the test for the left and right limb was analysed using custom-developed software (ARS-Dynamometry, S2P, Ljubljana, Slovenia; developed in Labview 8.1; National Instruments, Austin, TX, USA). Preliminary investigation on 20 male soccer players showed very high test-retest reliability of the hip adductor isometric test (CV = 5.5%; ICC = 0.92).

### 2.4. Data Analysis

Force signals were sampled at 1000 Hz and were transferred to a personal computer using a two-channel amplifier (InsAmp, Isotel, Logatec, Slovenia) and an analogue-to-digital card (NIUSB-6009, National Instruments, Austin, Texas, TX, USA). Force signals were preprocessed with a moving average filter using a 10 m window. The peak force values during the adductor test for left and right limb were analyzed using custom developed software (ARS-Dynamometry, S2P, Ljubljana, Slovenia; developed in Labview 8.1, National Instruments, Austin, Texas, TX, USA). Next, peak force values were multiplied by lever arm (leg length, in meter) to calculate hip adductor torque, and torque values were normalized to body mass (i.e., expressed in Nm per kg of body mass; Nm/kg). 

For the uninjured group, between-limb strength asymmetry in isometric hip adductor torque was calculated as a left:right limb ratio and, for the injured group, as an uninjured:injured limb ratio [28]. The between-limb strength asymmetry ratio was converted to a percentage difference using log-transformed raw data, followed by back transformation [28].

### 2.5. Statistical Analyses

All calculations were performed using SPSS for Windows (version 17.0; SPSS Inc, Chicago, IL, USA). Descriptive statistics (mean and SD) were calculated for age, anthropometric, and strength variables. Comparisons between injured and uninjured players in these variables were made using unpaired t tests. A paired-sample t-test was used to compare the normalized adductor strength of the affected and the unaffected side in the injured group. Cohen’s effects sizes (ES or *d*) for between-group differences were also calculated as follows [29]:

$$ES\left(d\right)=\frac{\left|mean2-mean1\right|}{SDpooled}$$

$$SDpooled=\frac{\sqrt{\left(n2-1\right)\cdot SD2^{2}+\left(n1-1\right)\cdot SD1^{2}}}{n1+n2-2}$$

where *mean*1 and *mean*2 are means of two groups, *SDpooled* is pooled standard deviation, *SD*1 and *SD*2 are standard deviations of two groups, and *n*1 and *n*2 are the sample sizes of the two groups. For within-group differences, ES was calculated as follows [29]:

$$ES\;\left(d\right)=\frac{\left|mean2-mean1\right|}{\sqrt{SD1^{2}+SD2^{2}-\left(2\cdot r\cdot SD1^{2}\cdot SD2^{2}\right)}}$$

where *mean1* and *mean2* are two means of a single group, *SD1* and *SD2* are two standard deviations of a single group, and *r* is the correlation between the two conditions.

Thresholds of 0.2, 0.5, and 0.8 for small, moderate, and large standardized differences in means, respectively, were used, as suggested by Cohen [29]. Binary logistic regression analysis was used to calculate odds ratios for groin injuries using age, previous injury (groin injury), isolated adductor strength, and between-limb strength asymmetry as covariates. A significance level of 0.05 was used for all tests.

## 3. Results

During the season, 10 clinically diagnosed groin injuries (no recurrences) were registered, of which seven were adductor-related, one iliopsoas-related, and two pubic-symphysis related. Groin injuries resulted in a mean of 14 days (range, 5–27 days) absence from full training and match play. The athletes who sustained groin injuries displayed no significant difference in age, height, and weight compared to uninjured players (all *p* > 0.05).

Pre-season strength performance for injured and uninjured soccer players is reported in Table 1. When compared to uninjured players, players who sustained a groin injury had significantly lower isometric adductor strength (t = 3.24; *p* = 0.002; mean difference = 0.79; standard error of difference = 0.25; 95% confidence interval of the difference = 0.30 to 1.29) and significantly higher between-limb adductor strength asymmetry (t = −2.45; *p* = 0.018; mean difference = −6.87; standard error of difference = 2.80; 95% confidence interval of the difference = −12.51 to −1.22) than uninjured players. The observed between-group differences in adductor strength performance were of moderate magnitude (ES = 0.83–1.05; see Table 1). In the injured group, the affected side was significantly weaker in the isometric adductor strength test than the unaffected side (2.43 Nm/kg vs. 2.87 Nm/kg; t = 7.85; *p* < 0.001; mean difference = 0.44; standard error of mean = 0.056; 95% confidence interval of the difference = 0.32 to 0.57; ES = 0.46).

The logistic regression model with groin injury as the dependent variable was statistically significant (χ^2^(4) = 15.75, *p* = 0.003). The model explained 45.2% of the variance in groin injury occurrence, and correctly classified 60% of cases. Players’ age (OR = 0.87; 95% CI: 0.65–1.16; *p* = 0.34), previous groin injury (OR = 4.41; 95% CI: 0.59–32.95; *p* = 0.148), and between-limb adductor strength asymmetry (OR = 1.11; 95% CI 0.98–1.26; *p* = 0.09) had no significant relationship with the incidence of future groin injury. In contrast, isometric adductor strength had a significant inverse relationship with the incidence of prospectively occurring groin injuries (OR = 0.19; 95% CI: 0.05–0.73; *p* = 0.016).

## 4. Discussion

The main findings of the present study were that (i) lower levels of isometric adductor strength significantly increased the risk of future groin injuries in professional soccer players, and (ii) the injured group had lower levels of isometric adductor strength and higher between-limb strength asymmetry vs. the uninjured group. These findings, together with limitations of the current study, are discussed in the following four sections.

### 4.1. Non-Modifiable Risk Factors

The first important finding of the present study was that non-modifiable risk factors, i.e., age and previous injury, were not significantly associated with an increased risk of future groin injuries. Previous research generally supports our results that age is not a significant risk factor for future groin injuries in soccer players [5,14,17,30]. In contrast to our results, previous reports [5,14,15,17,30] have generally found that previous injury is significantly associated with an increased propensity of future groin injury in soccer players. We believe that the main reason for this disparity is the methodological limitation of the current study. Namely, we observed an about four-fold increase in the probability of future groin injuries in players with injury history; however, due to the relatively low sample size, 95% CIs of those odds ratios included the value 1 (*p* = 0.15). Thus, due to this limitation of the current study, we cannot exclude previous groin injury as a risk factor for future groin injury in men’s soccer.

### 4.2. Adductor Strength and Between-Limb Strength Asymmetry as Modifiable Risk Factors

The most important finding of the present study was that lower levels of isometric adductor strength significantly increased the risk of future groin injuries in professional soccer players. Furthermore, the injured group had lower levels of isometric adductor strength vs. the uninjured group, and this difference was of a large magnitude. Finally, injured players had significantly weaker hip adductors of affected vs. unaffected limbs. Having in mind the high test-retest reliability and low within-individual variability of our testing protocol, we are confident of the precision of our measurements. Our findings compare well with the results from two previous prospective studies [14,16], suggesting that hip adductor weakness represents an important risk factor for groin injuries in men’s soccer. However, several authors failed to establish a significant association between isometric adductor strength and groin injury risk in soccer [12,17]. On the contrary, the authors of the later study reported that having higher than normal eccentric adduction strength was associated with an increased risk of groin injuries [17].

This discrepancy in results could, at least in part, be due to differences in adductor strength testing methodology. For example, the isometric adductor strength of the uninjured group in the current study (see Table 1) was higher than the eccentric adductor strength of uninjured professional soccer players in the study by Mosler et al. [17]. Given that the position of the dynamometer (externally fixed in the current study vs. handheld in the study by Mosler et al. [17]) and the normalization procedure were similar in both studies, and considering the phenomenon of bilateral strength deficit [31], these findings suggest that the manual handling of the dynamometer could underestimate player’s true adductor strength. Related to that, O’Brien et al. [27] recently reported that only 40–50% of the common variance exists between adductor strength measured with a handheld dynamometer and an externally fixed dynamometer similar to the one used in the current study. While our prospective study is not the first one that used an externally fixed dynamometer for testing soccer players’ adductor strength at the ankle level [13], no previous study has related this type of adductor strength measure with groin injury risk in soccer. Thus, bearing in mind the above, further prospective studies that also use an externally fixed dynamometer for testing adductor strength are needed to verify our findings.

The second important finding of this study was that higher levels of between-limb asymmetry in isometric adductor strength do not significantly increase the risk of subsequent groin injuries; although there was a trend towards the statistical significance (*p* = 0.09). Furthermore, the injured group had significantly higher between-limb strength asymmetry in isometric adductor strength compared with the uninjured group, and the observed group difference was also of large magnitude (see Table 1). The observed mean between-limb strength asymmetry in the injured group was 17%, i.e., somewhat higher than the frequently cited value of 15% that supposedly increases the risk of injuries [32]. These results generally suggest that a reduction in between-limb strength asymmetry in isometric adductor strength could be important in reducing the risk of incidence of future groin injuries. In contrast, Bourne et al. [32] recently reported very low (2.6%) between-limb strength asymmetry in isometric adductor strength of professional soccer players who sustained groin injury during the season. These findings, together with rather small odds ratio (1.16) for between-limb strength asymmetry observed in the current study, suggest that this factor has limited value in the prediction of future groin injuries. 

From a practical viewpoint, our results suggest that pre-season screening for adductor strength and strength asymmetry, as well as the strengthening of weak adductor muscles, could be of particular importance for the prevention of groin injuries in men’s soccer. Specifically, the adductor strength testing procedure used in the current study proved to be highly reliable and simple to use in professional soccer. Furthermore, to reduce the adductor weaknesses or strength asymmetries recorded during the screening process, and to address mechanisms of acute groin injuries [18], unilateral adductor strengthening exercises in both open and closed kinetic chain should preferably be used. Recent randomized controlled trials on semi-professional soccer players support this view, as the authors showed that simple unilateral adductor strengthening programs substantially reduced the prevalence and risk of groin problems in male soccer players [33].

### 4.3. Limitations

We acknowledge several limitations in this study. First, our sample size and, consequently, the recorded number of injury cases, were lower than recommended for detecting strong to moderate associations in prospective cohort studies [34]. Second, we did not record training and match exposure during the season. To decrease the influence of this factor, we included in the study only players who played more than 20 professional matches in the respective season [23,24]. Third, the assessment of adductor strength and between-limb strength asymmetry was only performed at a single time point at the start of the pre-season period. While previous prospective studies in soccer used similar approaches [12,13,14,15,16,17], we cannot exclude possible changes in strength over the pre-season and in-season periods. 

## 5. Conclusions

Our results indicate that professional soccer players with low isometric adductor strength (measured with an externally fixed dynamometer) are at an increased risk of groin injuries. Age, previous injury, and between-limb strength asymmetry were not significantly associated with an increased risk of groin injuries, although injured players had significantly higher between-limb strength asymmetry of adductor muscles than uninjured players. These results suggest that coaches should implement adductor strengthening into the training programs of soccer players, preferably using unilateral exercises to reduce between-limb strength asymmetry. Due to relatively low sample size in the current study, future prospective studies that use a similar strength testing methodology on professional soccer players are needed.

## Figures and Tables

**Table 1:** Pre-season adductor strength performance for injured and uninjured soccer players.

	Injured	Uninjured	Effect Size (*p*-Level)
Normalized isometric adductor strength (Nm/kg)	2.65 ± 1.06	3.44 ± 0.54	1.16 (0.002 *)
Between-limb adductor strength asymmetry (%)	16.82 ± 10.61	9.95 ± 6.88	0.88 (0.018 *)

* Statistically significant group difference a level of *p* < 0.05; Nm/kg—Newton-meter per kg of body mass.
